# Optimal doses of intranasal esketamine plus dexmedetomidine for sedating toddlers during transthoracic echocardiography: a prospective, double-blind, randomized trial

**DOI:** 10.1080/07853890.2025.2453087

**Published:** 2025-01-17

**Authors:** Dongjie Pei, Ting Xiao, Li Zeng, Siwei Wei, Lei Wang, Zhen Du, Shuangquan Qu

**Affiliations:** Department of Anesthesiology, Hunan Children’s Hospital, Changsha, Hunan, China

**Keywords:** Esketamine, sedation, pediatric, dexmedetomidine, transthoracic echocardiography

## Abstract

**Introduction:**

Esketamine has unique advantages in combination with dexmedetomidine for sedation in young children, owing to its sympathetic activity and mild respiratory depression. However, the optimal dose is yet to be determined. In this study, we compared the different doses of intranasal esketamine combined with dexmedetomidine for sedation during transthoracic echocardiography in toddlers.

**Patients and Methods:**

A total of 121 eligible children aged 13 years, who were scheduled for transthoracic echocardiography were randomized into three groups. They were treated with intranasal dexmedetomidine 1 mcg.kg^−1^ + esketamine 0.5 mg.kg^−1^ (group S1), dexmedetomidine 1 mcg.kg^−1^ + esketamine 1 mg.kg^−1^ (group S2), or dexmedetomidine 1 mcg.kg^−1^ + esketamine 1.5 mg.kg^−1^ (group S3). The primary outcome was the success rate of sedation, other outcomes included HR, SpO_2_, onset time, wake-up time, and adverse effects.

**Results:**

The success rate of sedation was significantly higher in groups S2 (85.4%) and S3 (87.5%) than ingroup S1 (60%) (*p* = 0.004). The baseline HR and SpO_2_ did not differ between the groups at the corresponding time points following drug administration. The onset time and duration of sedation in group S1 were significantly longer than those in groups S2 and S3 (*p* = 0.000). However, there were no differences in the wake-up time or adverse effects among the three groups.

**Conclusions:**

Intranasal administration of 1 mg.kg^−1^ esketamine combined with 1 mcg.kg^−1^ dexmedetomidine provided satisfactory sedation in young children undergoing transthoracic echocardiography. This sedative approach offers a rapid onset of awakening with few side effects.

**Clinical trial registration number:**

ChiCTR2200060976, 2022/06/14 (trail from August 2022 to January 2023)

## Introduction

Over the past few decades, the interest in painless or fearless examinations and therapies for children has increased. This has advanced the development of sedative drugs and techniques. Transthoracic echocardiography (TTE) is commonly used to diagnose cardiac illness in children. In the aftermath of the COVID-19 pandemic, several patients [1–2], particularly children with pre-existing conditions, may experience cardiac dysfunction. Therefore, they may need to undergo TTE more frequently to assess their cardiac function. However, the success of these examinations relies on the child’s cooperation. This is challenging for toddlers, and TTE typically requires sedation [3]. Drugs such as chloral hydrate, phenobarbital, midazolam, and sufentanil can be used for sedation in children. However, problems such as slow onset, long duration, and multiple side effects are common [4–6].

Dexmedetomidine is a highly selective α2-adrenergic receptor agonist widely used for sedation in children in many countries [7,8]. However, when used alone, dexmedetomidine may induce adverse circulatory effects [9,10], potentially rendering it unsuitable for children with heart disease. In contrast, esketamine, the S (+) enantiomer of ketamine, exhibits approximately twice the anesthetic and analgesic potency of racemic ketamine, coupled with fewer psychiatric side effects [6]. Additionally, it sustains protective airway reflexes post-use [11] and demonstrates sympathetic activity, countering the adverse circulatory effects of dexmedetomidine. Therefore, a combination of these two drugs may be suitable for sedation in children with heart disease. Importantly, these two drugs can be administered nasally. Intranasally administered drugs have pharmacokinetic features similar to those of intravenously administered drugs. This is because the nasal mucosa contains large blood vessels that allow rapid drug absorption, thereby preventing the first-pass elimination of oral medications[12–14]. As it is non-invasive and has a high level of acceptance, intranasal medication delivery is used more frequently to sedate children [15].

However, the appropriate dosage and potential adverse effects of intranasal esketamine combined with dexmedetomidine for sedating young children have not yet been reported. Therefore, this study aimed to compare the efficacy and safety of different doses of intranasal esketamine combined with dexmedetomidine in toddlers. This study is valuable in promoting sedation in children and provides a reference for clinical practice.

## Patients and methods

This prospective, double-blind, randomized trial was registered on the Chinese Clinical Trials website (ChiCTR2200060976, 2022/06/14) and approved by the Hunan Children’s Hospital Medical Ethics Committee (HCHLL-2021-66, 2021/09/02). This research was supported by the Scientific Research Fund of the Hunan Medical Association (HNA202101020) and the Scientific Research Project of the Hunan Provincial Health Commission (D202304118023).

### Study design and participants

Following parental signatures on an informed consent form, children aged 1–3 years who were scheduled for TTE with ASA classes I–II were enrolled in this study. The exclusion criteria included refusal to participate in the study, cyanotic congenital heart disease, allergy to medications, structural anomalies of the nose and airway, inherited metabolic disease, organ dysfunction, intracranial and intraocular hypertension, and weight >20 kg.

**Figure 1.**
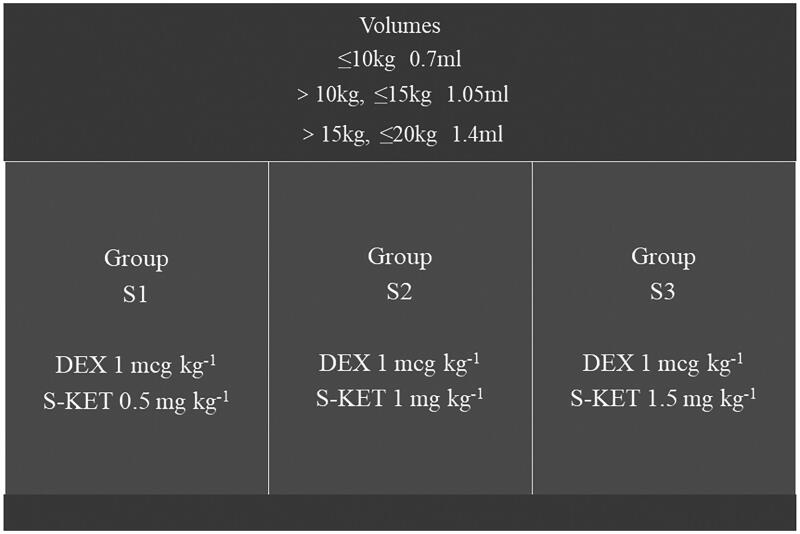
Drug volume of different body weights and drug dosage of different groups. DEX: dexmedetomidine; S-KET: esketamine.

Children were randomly divided into three groups, with an equal number of children in each group: dexmedetomidine 1 mcg.kg^−1^ + esketamine 0.5 mg.kg^−1^ (group S1), dexmedetomidine 1 mcg.kg^−1^ + esketamine 1 mg.kg^−1^ (group S2), and dexmedetomidine 1 mcg.kg^−1^ + esketamine 1.5 mg.kg^−1^ (group S3). The investigator prepared the sedative medications. The drug concentrations were 25 mg/ml esketamine and 100 mcg/ml dexmedetomidine, diluted with 0.9% sodium chloride. The volumes of intranasal drugs were 0.7 (weight ≤ 10 kg), 1.05 (weight > 10 kg, ≤ 15 kg), and 1.4 mL (weight > 15 kg, ≤ 20 kg), respectively. The drug volumes for different body weights and doses in different groups are shown in Figure 1. We excluded children weighing > 20 kg in consideration of drug concentration and the practicality of intranasal administration. The anesthesiologists responsible for sedation, sonographers, children, and their parents were blinded to the group assignment.

### Sedation protocol

Before administering the medication, the nasal canal was cleaned, and the children were instructed to refrain from consuming solid food and milk for 6 h, breast milk for4 h, and clear liquids for 2 h. The children were held by their parents in the supine position and individually dosed into both nostrils using a mucosal nebulizer (MAD Nasal Teleflex). The baseline heart rate (HR), blood pressure (BP), and oxygen saturation (SpO2) of the children were noted. All aforementioned vital signs, except BP, were measured every five minutes for the first 30 min following drug administration. Due to the potential for waking up the children, BP was not measured again after administration, as non-invasive BP cuff inflation can be disruptive. A modified Ramsey score (Table 1) was used. Instead of tapping the forehead, a response to the probe placement on the body was used[3, 16]. TTE was initiated when the level of sedation was ≥ 4 [17]. In the event of sedation failure, defined as failure to reach the desired level after 45 min of administration, a rescue dose of 1 mcg.kg^−1^ dexmedetomidine was administered to the child. If physical movement during the examination necessitated interruption, the child was administered a supplementary dose of 1 mcg.kg^−1^ dexmedetomidine. Toddlers were transferred to the recovery room post examination, allowing them to awaken naturally. The discharge criteria were met when the Aldrete score was ≥ 9 points [18].

**Table 1.** Modified Ramsay Scale.

Sedation Scale	Responses
1	Anxious or/and agitated
2	Co-operative, oriented and tranquil
3	Responds to commands only
4	A brisk response to TTE probe placement
5	A sluggish response to TTE probe placement
6	No response

TTE: Transthoracic echocardiography.

### Study outcomes

The primary outcome was the success rate of sedation. The onset time, wake-up time, examination time, total sedation time, and side effects after drug administration were recorded. Onset time was defined as the interval between the time the drug was administered and a Ramsay score ≥ 4 points. The onset time for children who received the rescue dose was 45 min. Wake-up time refers to the period from when the child’s Ramsay score ≥ 4 points to spontaneous awakening. The total sedation time was the duration from when the child was administered the sedative until discharge.

### Statistical analysis

Based on our previous clinical observation, the success rate of sedation using 1 mcg.kg^−1^ dexmedetomidine combined with 0.5 mg.kg^−1^ esketamine intranasal was approximately 65%. The sample size was calculated using PASS V15 software. The cutoff for non-inferiority was 15%, α = 0.05, β = 0.2, with a 10% miss rate. This calculation resulted in a combined sample size of 114 patients, with an average of 38 patients per group.

Data were analyzed using SPSS v19.0 software. Measurement data were presented as medians (quartiles), and counting data were presented as rates (%). Continuous variables were tested for normality using the Kolmogorov–Smirnov test and comparisons of data that conformed to a normal distribution were performed using one-way ANOVA, with non-normal distributions using the Kruskal-Wallis test. The χ^2^ test or Fisher’s exact test was used for categorical variables. *p* < 0.05 was considered statistically significant.

## Results

**Figure 2.**
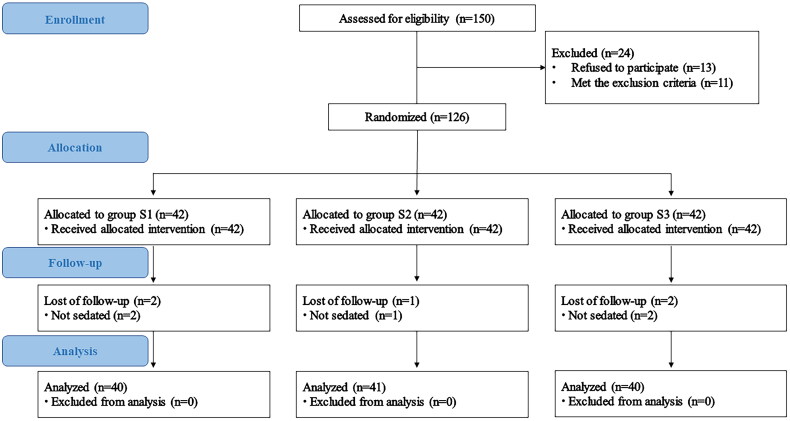
Flow chart.

In total, 150 children who underwent TTE at our hospital between August 2022 and January 2023 were recruited for this study. Of these, 11 were excluded because they did not meet the inclusion criteria, 13 parents declined to participate, and 126 children were randomly assigned. Due to their inability to be sedated, two children from group S1, one from group S2, and two from group S3 were excluded. Consequently, the statistical analysis included 121 children: 40 in group S1, 41 in group S2, and 40 in group S3. Figure 2 illustrates the flow of the trail, and Table 2 provides a list of disease classifications for children.

**Table 2.** Classification of diseases.

Diseases	Count (*n* = 121)
Ventricular septal defect	43
Atrial septal defect	30
Patent ductus arteriosus	28
Kawasaki disease	10
Rheumatic fever	5
Coarctation of aorta	3
Pulmonary valve coarctation	2

The three groups showed no statistically significant differences in the general characteristics of the children (Table 3).

**Table 3.** Demographic parameters.

	Group S1 (*n* = 40)	Group S2 (*n* = 41)	Group S3 (*n* = 40)	*P*
Sex (male/female)	21/19 (52.5%;47.5%)	26/15 (63.4%;36.6%)	27/13 (67.5%;32.5%)	0.363
Age (months)	25.00 (18.25-31.75)	24.00 (16.00-31.00)	21.50 (17.00-24.75)	0.117
Weight (kg)	11.75 (10.50-13.25)	11.70 (10.00-13.00)	11.25 (10.00-12.65)	0.822

Data were expressed as median (quartiles).

The success rates in groups S1, S2, and S3 were 60%, 85.4%, and 87.5%, respectively (Table 4). While there was no statistical difference between groups S2 and S3, the success rates in both groups were significantly higher than those in group S1.

**Table 4.** Success rates.

	Success rates
Group S1 (*n* = 40)	24 (60%)
Group S2 (*n* = 41)	35 (85.4%)
Group S3 (*n* = 40)	35 (87.5%)
*P*	0.004

**Figure 3.**
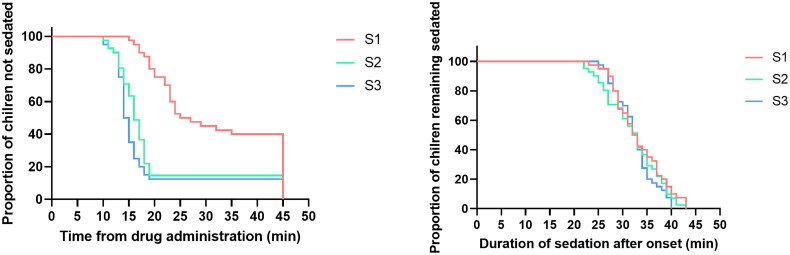
Kaplan–Meier curves for the sedation onset time after administration in minutes (left), and the duration of sedation after onset in minutes (right).

Compared with groups S2 and S3, the onset time of group S1 was much longer. However, there was no significant difference between groups S2 and S3. in addition, the total sedation time in group S1 was longer than that in groups S2 and S3 (Figure 3). The three groups showed no significant differences in the wake-up or examination times. (Table 5)

**Table 5.** Sedation time (min).

	Group S1 (*n* = 40)	Group S2 (*n* = 41)	Group S3 (*n* = 40)	*P*
Onset time	26.00 (20.50–45.00)	16.00 (14.00–18.00)	14.50 (13.25–16.75)	0.000
Wake-up time	32.50 (29.00–37.00)	33.00 (27.00–37.00)	32.50 (29.00–35.00)	0.745
Examination time	16.50 (14.00–19.75)	16.00 (14.00–19.50)	18.00 (13.00–21.00)	0.663
Total time	62.00 (55.00–74.00)	49.00 (44.00–53.00)	47.50 (45.25–52.00)	0.000

Data were expressed as median (quartiles).

**Figure 4.**
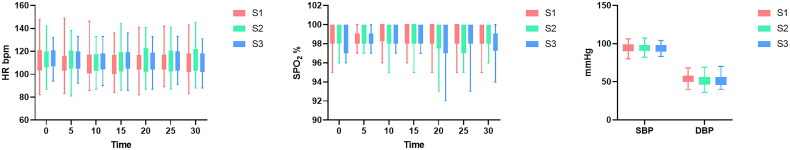
Vital signs of three groups.

Baseline HR, BP, and SpO2 did not differ among the three groups. However, following administration, the HR and SpO2 showed statistically significant differences among the three groups at the corresponding time points. Notably, after medication administration, the HR and SpO2 in all groups did not change significantly from baseline (Figure 4).

The adverse events are summarized in Table 6. One child in group S3 exhibited symptoms of upper airway obstruction caused by tonsillar hypertrophy, which was alleviated when the child held their jaw. In four patients, oxygen desaturation was recorded, with a minimum level of 92%. However, these children recovered without oxygen inhalation. After minimal stimulation, the HR returned to normal in four children experiencing bradycardia (< 20% of baseline).

**Table 6.** Adverse events.

Adverse events	Group S1 (*n* = 40)	Group S2 (*n* = 41)	Group S3 (*n* = 40)	*P*
Nausea	3	2	3	0.861
Increased secretions	2	2	1	0.819
SpO2 < 95%	0	1	3	0.162
Upper airway obstruction	0	0	1	0.363
Bradycardia	1	1	2	0.766
Delirium	0	0	0	

## Discussion

According to our findings, the intranasal administration of 1 mg.kg^−1^ of esketamine and 1 mcg.kg^−1^ of dexmedetomidine provided satisfactory sedation in young children undergoing TTE. This approach offers quick onset and wake-up times, with fewer side effects.

Most pediatric examinations require sedation, and various administration routes, including intravenous, oral, and rectal, are commonly employed. Intravenous administration is invasive and often frightens children. Oral administration is associated with low bioavailability and unreliable efficacy. Rectal stimulation is common, and many children experience diarrhea post-administration.

In this study, we used a mucosal nebulizer for nasal administration, which resulted in minimal discomfort and high bioavailability [19]. This method is particularly suitable for pediatric patients.

Ideally sedatives should not induce respiratory depression, have a rapid onset and recovery, and have entail minimal side effects. Several medications, including ketamine, dexmedetomidine, midazolam, and chloral hydrate, can be used to sedate children. Chloral hydrate was previously the most widely used sedative drug with a sedation success rate of 60%–92.8%. However, its use has been restricted owing to frequent side effects, such as vomiting, drowsiness, and delayed sedation, which can lead to unintended outcomes. Midazolam is associated with a faster onset and recovery from sedation. However, it is associated with a higher rate of sedation failure and adverse events [20].

Additionally, concomitant co-administration of benzodiazepines and ketamine is a risk factor for airway complications in children [21]. Therefore, dexmedetomidine is a suitable sedative for children. It acts on the locus coeruleus and induces sleepiness, similar to natural sleep, without causing respiratory depression. However, it can lead to bradycardia and hypotension in young children [6]. Ketamine exhibits mild bronchiectasis and sympathomimetic effects [22], maintains the hypercapnia respiratory reflex, and does not cause respiratory depression, particularly in children with asthma.

Esketamine is a novel N-methyl-D-aspartate receptor inhibitor with significant sedative and analgesic properties. It is twice as effective as racemic ketamine and has fewer psychiatric side effects [23]. Numerous studies have demonstrated that preoperative intranasal administration of combined dexmedetomidine and ketamine in children is of pharmacological interest, as both drugs have opposing hemodynamic effects. The onset time is shorter and there is less postoperative agitation and secretion compared with single agents [6, 24].

Liu et al.’ confirmed that 0.5 mg.kg^−1^ of esketamine can relieve separation anxiety before pediatric strabismus surgery [25]. However, following intranasal administration of dexmedetomidine at doses of 1 and 2 mcg.kg^−1^, no significant difference in sedation satisfaction was observed among children aged 1–4 years [26]. This is based on the fact that the initial doses of esketamine and dexmedetomidine in our study were 0.5 mg.kg^−1^ and 1 mcg.kg^−1^.

Our findings revealed a sedation success rate of only 60% when administering 1 mcg.kg^−1^ of dexmedetomidine and 0.5 mg.kg^−1^ of esketamine concurrently. Ola M. Zanaty et al.’ discovered that combining nebulization dexmedetomidine at 1 mcg.kg^−1^ and ketamine at 1 mg.kg^−1^ as a premedication in children aged 3–6 years achieved satisfactory sedation [27]. Given that of the potency of esketamine is twice that of racemic ketamine, its dosage was similar to our initial dosage. This difference may be explained by variations in drug absorption resulting from the different modes of administration, such as nasal administration and nebulization. Additionally, evidence suggest that younger children generally require higher drug doses to achieve comparable levels of sedation owing to pharmacokinetic factors [28].

When administered intranasally alone at a dose of 2 mg.kg^−1^, esketamine took 18 ± 13 min to reach peak plasma concentration [29]. However, in our study, the onset time was shorter in the S2 and S3 groups than that previously reported. This difference in outcomes may be related to the definition of the onset time, dexmedetomidine may accelerate the onset time of ketamine sedation [30]. The intranasal administration of 6 mg.kg^−1^ ketamine reduced the onset time to 5.79 ± 1.42 min, achieving a sedation rate of 93% [31]. However, we opted not to use an equivalent dose of 3 mg.kg^−1^ esketamine in our study. The decision was based on concerns that it would increase the volume of intranasal administration, causing discomfort to the children. Additionally, unlike in previous studies, our participants did not undergo invasive procedures, reducing the need for a deeper level of sedation, as reported in earlier studies. Another study demonstrated that intranasal administration of dexmedetomidine and ketamine prolonged recovery compared with dexmedetomidine alone [7]. Furthermore, oral preoperative administration of ketamine at higher dosages was found to slow recovery [32]. Because we did not establish a separate control group for dexmedetomidine, we could not determine whether the combination of dexmedetomidine and esketamine resulted in a prolonged recovery time. However, at the dose used in this study, an increased amount of esketamine had no noticeable effect on the children’s wake-up time.

With the exception of one child in group S3 who experienced upper airway obstruction due to tonsillar hypertrophy and required management, the vital signs of the children remained stable. Although there was no statistically significant difference between groups S3 and S1 or S2, we observed an increase in the number of children with SpO_2_ < 95%. Ketamine does not conform to the typical dose-response sedation continuum and ketamine has a dissociation threshold. Once this threshold is exceeded, additional doses of ketamine do not increase the risk of respiratory depression, unlike opioids and inhalational anesthetics [33–34]. Therefore, we believe that this phenomenon may not explain the risk of respiratory depression associated with higher esketamine doses, and further observation with a large sample size may be necessary for confirmation.

Following the intravenous administration of esketamine, Liu reported a dose-dependent increase in HR [35]. This contrasts with our finding that the HR in each group did not differ significantly from the baseline following drug administration. This may be related to the administration of dexmedetomidine, which blocks the excitatory sympathetic effects of esketamine [36]. However, it has also been demonstrated that intranasal administration of esketamine at 2 mg.kg^−1^ does not produce clinically detectable sympathomimetic effects [37].

Up to 35.3% of individuals experience nausea as a side effect after receiving intranasal ketamine [38]. After nebulizer administration, the incidence can be reduced by half [39]. In our investigation, there was a low incidence of nausea and vomiting in all three groups, which is consistent with a study conducted by Yang et al. [40]. Increased ketamine secretion is another common side effect of ketamine. However, they occur less frequently with esketamine than with ketamine [41]. Additionally, dexmedetomidine-induced xerostomia can help reduce these secretions [30]. These findings are consistent with this study.

This study had certain limitations. First, because dexmedetomidine alone was not used as a control, the onset time and side effects of the combination could not be compared. Second, since children with cyanotic cardiac conditions were excluded, it was impossible to determine the safety and effectiveness of sedation with dexmedetomidine and esketamine. Third, adverse events were observed until hospital discharge but they occurred later in some patients, suggesting that further observation in future studies is warranted.

## Conclusions

When administered intranasally, the combination of dexmedetomidine 1 mcg.kg^−1^ and esketamine 1 mg.kg^−1^ provided adequate sedation, rapid onset and recovery times, and low occurrence of side effects in toddlers undergoing TTE.

## Supplementary Material

CONSORT_2010_Checklist.doc

## Data Availability

The data that support the findings of this study are available from the corresponding author upon reasonable request.
